# Early Prognostication in Pediatric Severe Traumatic Brain Injury in South America: Development of a Local Pediatric-Specific Model and Validation of Established Models

**DOI:** 10.1089/neur.2024.0157

**Published:** 2025-02-19

**Authors:** Madeline E. Greil, Omar Abdelmaksoud, Lauren L. Agoubi, Julia Velonjara, Jin Wang, Gustavo Petroni, Silvia Lujan, Nahuel Guadagnoli, Michael J. Bell, Monica S. Vavilala, Robert H. Bonow, the PEGASUS Argentina Study Group

**Affiliations:** ^1^ Department of Neurological Surgery, Harborview Medical Center, Seattle, Washington, USA.; ^2^ Department of Surgery, School of Medicine, University of Washington, Seattle, Washington, USA.; ^3^ Harborview Injury Prevention and Research Center, University of Washington, Seattle, Washington, USA.; ^4^ Department of Anesthesiology and Pain Medicine, School of Medicine, University of Washington, Seattle, Washington, USA.; ^5^ Department of Pediatrics, School of Medicine, University of Washington, Seattle, Washington, USA.; ^6^ Centro de Informática e Investigación Clínica, Rosario, Argentina.; ^7^ Care Medicine, Children’s National Hospital, Washington, District of Columbia, USA.

**Keywords:** pediatric, prognosis, South America, traumatic brain injury

## Abstract

Prognostication in severe traumatic brain injury (sTBI) is important, but few models are pediatric-specific and from low- and middle-income countries where head computed tomography (CT) scans may not be routinely available. We assessed intensive care unit admission risk factors for early mortality and unfavorable outcome in a secondary analysis of 115 children (mean 7.0 years, standard deviation [sd] 5.3) receiving sTBI (Glasgow Coma Scale [GCS] total score ≤8 or GCS motor ≤5) care in South America who participated in the 16 hospital Pediatric Guideline Adherence and Outcomes (PEGASUS) Argentina trial between September 1, 2019, and July 13, 2020. Outcomes were 14-day mortality and 3-month Glasgow Outcome Scale-Extended for Pediatrics (GOS-E Peds). First, we examined univariate associations of predictors with the two outcomes. Then, two PEGASUS logistic regression models (core model with only clinical variables and full model with both clinical and CT variables) for each of the outcomes were derived. Models were examined for fit and compared for prediction. The locally derived PEGASUS model shows a good core prediction of 14-day (area under the receiver operating characteristic curve [AUROC]: 0.92; confidence interval [CI]: 0.85–0.99) and 3-month (AUROC 0.82 CI 0.73–0.91) outcomes; findings are similar to the International Mission on Prognosis and Analysis of Randomized Controlled Trials in TBI (IMPACT), Corticosteroid Randomization after Significant Head Injury (CRASH), and Petroni models. There was no difference between core and full models in prognosticating 14-day mortality, but IMPACT (*p* = 0.01) and PEGASUS (*p* = 0.01) full models outperformed their respective core models for 3-month GOS-E Peds. Core models, including PEGASUS, can be used but full models are preferred to prognosticate outcomes after pediatric sTBI in South America. PEGASUS model validation against external datasets is needed.

## Introduction

Traumatic brain injury (TBI) affects 69 million individuals annually and is the leading global traumatic cause of death and disability particularly in low- and middle-income countries (LMICs).^1^ Survivors of severe TBI (sTBI) experience significant disability.^2,3^ On a per-patient basis, the magnitude of these effects is amplified among children, in part due to greater life expectancy, and predicting outcomes in pediatric sTBI is important.

Prognostic estimations can inform conversations with families and help to guide triage, resource allocation, and treatment decisions in acute care, as well as facilitate comparison of outcomes across study samples and stratification of patients included in randomized trials.^3,4^ Two commonly utilized TBI prognostic models include the International Mission on Prognosis and Analysis of Randomized Controlled Trials in TBI (IMPACT) and the Medical Research Council Corticosteroid Randomization after Significant Head Injury (CRASH) models but mostly included older children and adults.^4,5^ The CRASH model is derived from adults patients in both high-income countries (HICs) and LMICs^6^ and IMPACT included adults with moderate TBI. Although Petroni et al. assessed the utility of these predictors in South America, this cohort also included only teenage and adult sTBI patients.^7^ Because none of these models were derived from a pediatric-specific sTBI cohort in South America, it is unclear if these models should be used for prognostication in pediatric sTBI in South America. It has been suggested that both CRASH and IMPACT models should not be used in routine clinical practice due to model age that may overpredict poor outcomes, poor model fit that worsens over time, and large variance in outcomes, suggesting a need for updating prognostic models for pediatric sTBI.^8,9^

Prior validation of established models shows better model performance when applied to cohorts similar to those used to derive them since differences in social, cultural, and economic factors can lead to relative under-performance.^4,6,7^ Age influences survival and neurological recovery post-TBI and young children have unique anatomy and physiology, but information on young children is extrapolated from adult studies and data from LMICs is lacking.^10,11^ None of the major prognostic tools for TBI included young children in their derivation cohorts. Leveraging existing data from the Pediatric Guideline Adherence and Outcomes (PEGASUS) pediatric sTBI study,^12^ we evaluated if a locally derived pediatric-specific PEGASUS model with clinical, or clinical and computed tomography (CT) variables at intensive care unit (ICU) admission prognosticates 14-day and 3-month outcomes. We also validated select established models with PEGASUS data.

## Methods

### PEGASUS project

The PEGASUS project involved 16 hospitals: 14 in Argentina, 1 in Chile, and 1 in Paraguay. All hospitals are government-administered tertiary care facilities with pediatric ICUs. Ten hospitals are solely dedicated to pediatric care, whereas six have both pediatric and adult ICUs. Each study hospital has a principal investigator who prospectively enrolled patients meeting eligibility criteria. This work was approved by the institutional review boards of the 16 PEGASUS study hospitals, and written informed consent was obtained in Spanish. The University of Washington Institutional Review Board approved this study as minimal risk.

### Study design and patient population

We performed a secondary analysis of prospectively collected observational usual care PEGASUS data between September 1, 2019, and July 13, 2020. Patients were included if they were admitted to ICU for sTBI care, which required Glasgow Coma Scale (GCS) score ≤8 or, if tracheally intubated, GCS motor ≤5, and either a mechanism of injury or CT scan indicating TBI. Patients who were admitted to hospital with GCS >8 but later deteriorated and required sTBI care in ICU were included at the time of deterioration assessment. We used admission GCS motor because the study goal was early prognostication. Clinicians assessed and recorded GCS as part of clinical care. Patients were excluded if missing both outcome variables.

### Data collection

Data on age, GCS motor, worst pupillary response (none reactive, one reactive, or both reactive), and maximum nonhead Abbreviated Injury Scale (AIS) score were abstracted from the study database.

### Outcomes

Main outcomes were: (1) 14-day mortality and (2) 3-month Glasgow Outcome Scale-Extended for Pediatrics (GOS-E Peds) scores, dichotomized as favorable (GOS-E Peds scores 5–8) or unfavorable (GOS-E Peds scores 1–4). The 14-day mortality was selected because they are used in established models (CRASH and IMPACT). We used the 3-month GOS-E Peds because we had those data available as part of the study. The site PI or coordinator contacted parents/guardians by either telephone or visit.

### Predictors

Predictors were age, sex, injury characteristics, any hypoxia or hypotension during ICU day 1, and classification of first CT.

#### Hypotension and hypoxia definitions

Hypotension was defined as a systolic blood pressure (SBP) <70 mmHg + 2*age (in years) or SBP <90 mmHg if age >12 years, and hypoxia was defined as oxygen saturation level of <90% during ICU day 1.

#### CT classification system

**FIG. 1.**
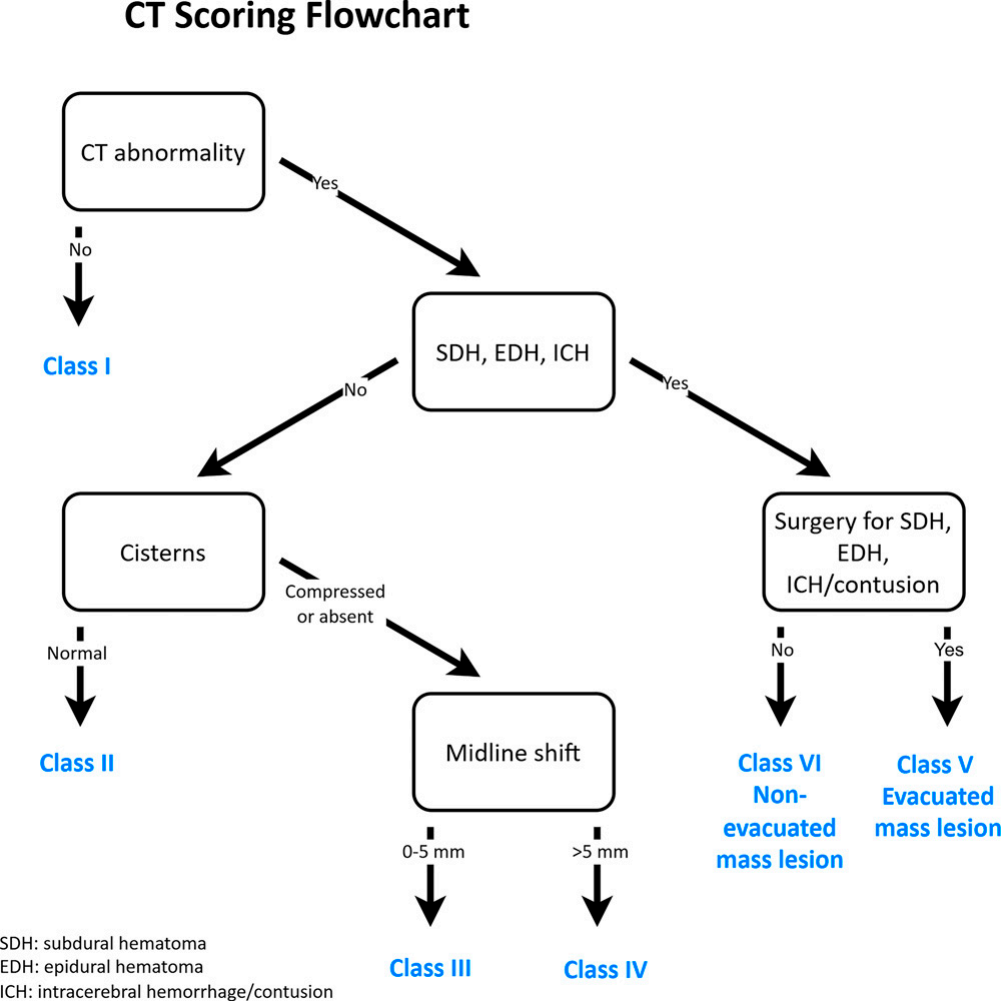
Modified Marshall CT classification system used in PEGASUS prognostic model development. CT, computed tomography; PEGASUS, Pediatric Guideline Adherence and Outcomes.

CT findings were abstracted from radiology report entries as abnormal versus normal CT, compressed or absent basal cisterns, obliteration of the third ventricle or basal cistern, and the presence or absence of midline shift >5 mm, epidural hematoma (EDH), subdural hematoma (SDH), intracerebral hemorrhage (ICH)/contusion, and subarachnoid hemorrhage. This information was used to classify scans into an abstracted Marshall score as described. As this was a secondary analysis of previously collected data, we did not have original CT scan images or all data points necessary to classify scans according to the Marshall classification.^13^ Therefore, we used accessible data points to extrapolate the classification. As shown in Figure 1, our procedure was as follows: first, any CT scan marked as without abnormality was assigned Class I. For a CT scan marked as having an SDH, EDH, or ICH, patients were classified as either nonevacuated mass lesion (Class VI) or evacuated mass lesion (Class V) based on whether the patient was marked as having undergone surgery for an SDH, EDH, contusion, or ICH. For those without an SDH, EDH, or ICH, we next looked at the classification of the cisterns. If they were normal, the scan was classified as Class II. If the cisterns were compressed or absent, patients were classified as Class III if they had ≤5 mm of midline shift and Class IV if they had >5 mm midline shift.

### Statistical analysis

Since many LMICs may not have readily available CT scanning at admission, we defined “core” PEGASUS model as having only clinical variables and the “full” PEGASUS model to have both clinical and CT variables. The three other models (IMPACT,^6^ CRASH,^4^ and Petroni^7^) also have versions of a “core” and “full” model but use different terms and include different variables to define these terms.

#### PEGASUS model

To build the PEGASUS model, we first examined univariate associations of ICU admission predictors with 14-day mortality and unfavorable 3-month GOS-E Peds using Chi-square statistics for categorical variables and Student *t-*test statistics for continuous variables. Fisher’s exact test was used for variables with a small sample size. Logistic regression with backward elimination was used to model the relationship between predictors with *p* < 0.2 in the univariate test and the two outcomes. Final logistic regression models were created with variables retained if they were significant at *p* < 0.05. Age was included in the core and full models regardless of significance because of the pediatric population of interest.

Odds ratios and 95% confidence intervals (CIs) were calculated in reference to “no” for binary variables. For additional categorical variables, reference categories were as follows: admission GCS motor 4–6, bilateral reactive pupils, and CT Marshall Class I or II (normal CT head or diffuse injury with patent cisterns and 0–5 mm midline shift).

#### Validating established models with PEGASUS data

We used the published coefficients from the CRASH,^6^ IMPACT,^4^ and Petroni et al.^7^ models to fit available PEGASUS data. We validated both core and full IMPACT and Petroni models against PEGASUS data but because CT evidence of petechial hemorrhages was not recorded in the PEGASUS study, we only validated the core CRASH model.

#### Comparison of models

Model fit and area under the receiver operating characteristic curve (AUROC) were assessed for each model (IMPACT, CRASH, Petroni, and PEGASUS). AUROC was compared between the four models for each of the two outcomes using the roccomp function in STATA. STATA Statistical Software Release 16 (StataCorp LLC, College Station, TX, USA) was used for all analyses.

## Results

**FIG. 2.**
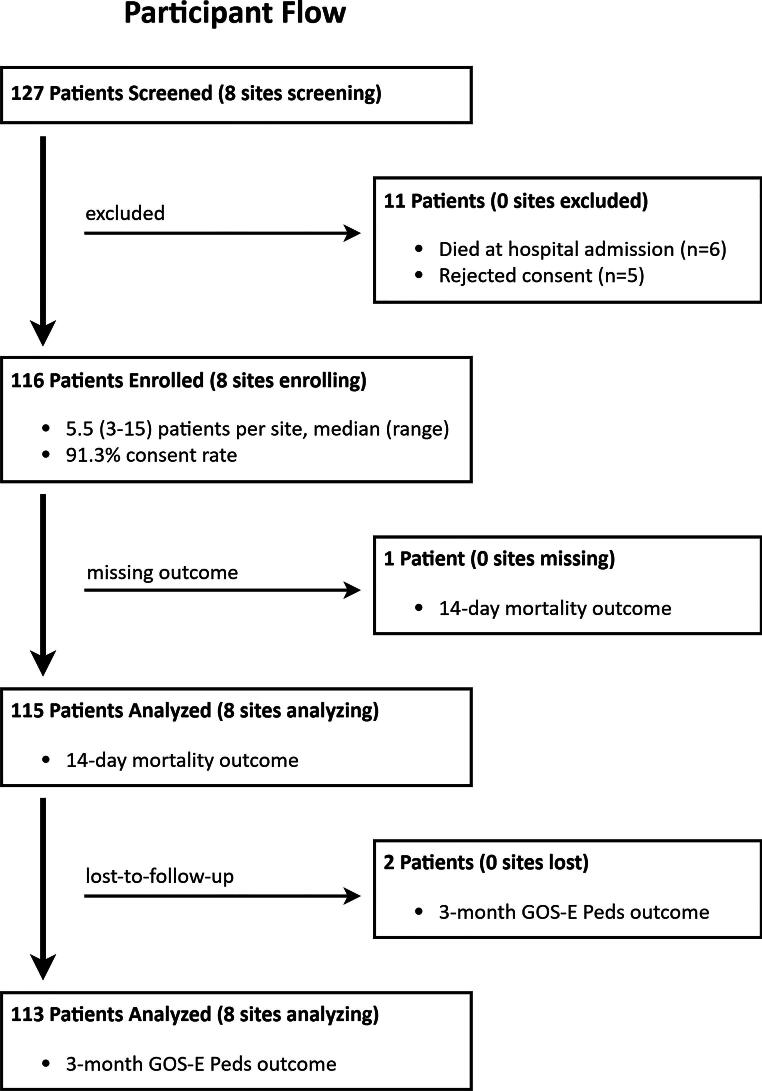
Participant flow diagram. GOS-E Peds, Glasgow Outcome Scale-Extended for Pediatrics.

Data from 115 children 7.0 ± 5.3 years were analyzed; one patient missing both outcomes of interest was excluded (Fig. 2); 73.0% of patients had GCS total ≤8 or GCS motor ≤5 at admission, while the remainder deteriorated in hospital to qualify for inclusion. Approximately one-third (35.7%) of patients had admission GCS motor 1, indicating no movement to stimulation (Table 1). The max nonhead AIS was <3 in two-thirds (66.1%) of patients. More than half of the patients (58.4%) had two reactive pupils.

**Table 1.** Patient and Clinical Characteristics

	14-day mortality				3-month outcome (GOS-E Peds)			
	Total *N* (%)	Alive *N* (%)	Dead *N* (%)		Total *N* (%)	Favorable *N* (%)	Unfavorable *N* (%)	
Predictor	*N* = 115	*N* = 106	*N* = 9	*p*	*N* = 113	*N* = 86	*N* = 27	*p*
Age, mean (SD) in years	7.0 (5.3)	7.0 (5.3)	7.4 (5.4)	0.83	7.0 (5.2)	6.9 (5.1)	7.3 (5.8)	0.71
Max nonhead AIS				1				0.17
<3	76 (66.1)	70 (66.0)	6 (66.7)		75 (66.4)	60 (69.8)	15 (55.6)	
≥3	39 (33.9)	36 (34.0)	3 (33.3)		38 (33.6)	26 (30.2)	12 (44.4)	
Admission GCS motor				**0.001**				**0.01**
1	41 (35.7)	37 (34.9)	4 (44.4)		41 (36.3)	28 (32.6)	13 (48.2)	
2	5 (4.4)	2 (1.9)	3 (33.3)		4 (3.5)	1 (1.2)	3 (11.1)	
3	5 (4.4)	5 (4.7)	0 (0.0)		5 (4.4)	3 (3.5)	2 (7.4)	
4	21 (18.3)	20 (18.9)	1 (11.1)		20 (17.7)	15 (17.4)	5 (18.5)	
5 or 6	27 (23.5)	27 (25.5)	0 (0.0)		27 (23.9)	27 (31.4)	0 (0.0)	
Unknown	16 (13.9)	15 (14.2)	1 (11.1)		16 (14.2)	12 (14.0)	4 (14.8)	
Worst pupillary response				**0.002**				0.14
Nonreactive	7 (6.1)	4 (3.8)	3 (33.3)		6 (5.3)	3 (3.5)	3 (11.1)	
One reactive	6 (5.2)	5 (4.7)	1 (11.1)		6 (5.3)	3 (3.5)	3 (11.1)	
Both reactive	67 (58.3)	65 (61.3)	2 (22.2)		66 (58.4)	51 (59.3)	15 (55.6)	
Unknown	35 (30.4)	32 (30.2)	3 (33.3)		35 (31.0)	29 (33.7)	6 (22.2)	
Admission or ICU Day 1 hypoxia				0.54				0.46
No	81 (70.4)	76 (71.7)	5 (55.6)		80 (70.8)	63 (73.3)	17 (63.0)	
Yes	33 (28.7)	29 (27.4)	4 (44.4)		32 (28.3)	22 (25.6)	10 (37.0)	
Unknown	1 (0.9)	1 (0.9)	0 (0.0)		1 (0.9)	1 (1.2)	0 (0.0)	
Admission or ICU Day 1 hypotension				**0.05**				**0.005**
No	57 (49.6)	56 (52.8)	1 (11.1)		55 (48.7)	49 (57.0)	6 (22.2)	
Yes	57 (49.6)	49 (46.2)	8 (88.9)		57 (50.4)	36 (41.9)	21 (77.8)	
Unknown	1 (0.9)	1 (0.9)	0 (0.0)		1 (0.9)	1 (1.2)	0 (0.0)	
CT result				1				0.57
Normal	4 (3.5)	4 (3.8)	0 (0.0)		4 (3.5)	4 (4.7)	0 (0.0)	
Abnormal	111 (96.5)	102 (96.2)	9 (100.0)		109 (96.5)	82 (95.3)	27 (100.0)	
Basal cisterns				**0.002**				**0.01**
Normal	82 (71.3)	80 (75.5)	2 (22.2)		80 (70.8)	67 (77.9)	13 (48.2)	
Compressed/Absent	31 (27.0)	24 (22.6)	7 (77.8)		31 (27.4)	18 (20.9)	13 (48.2)	
Unknown	2 (1.7)	2 (1.9)	0 (0.0)		2 (1.8)	1 (1.2)	1 (3.7)	
Midline shift >5mm				0.49				0.19
No	94 (81.7)	87 (82.1)	7 (77.8)		92 (81.4)	72 (83.7)	20 (74.1)	
Yes	14 (12.2)	12 (11.3)	2 (22.2)		14 (12.4)	8 (9.3)	6 (22.2)	
Unknown	7 (6.1)	7 (6.6)	0 (0.0)		7 (6.2)	6 (7.0)	1 (3.7)	
Epidural hematoma				0.06				**0.01**
No	80 (69.6)	71 (67.0)	9 (100.0)		78 (69.0)	54 (62.8)	24 (88.9)	
Yes	35 (30.4)	35 (33.0)	0 (0.0)		35 (31.0)	32 (37.2)	3 (11.1)	
Unknown	0 (0)	0 (0)	0 (0)		0 (0)	0 (0)	0 (0)	
Subdural hematoma				0.45				**0.005**
No	81 (70.4)	76 (71.7)	5 (55.6)		79 (69.9)	66 (76.7)	13 (48.2)	
Yes	34 (29.6)	30 (28.3)	4 (44.4)		34 (30.1)	20 (23.3)	14 (51.8)	
Unknown	0 (0)	0 (0)	0 (0)		0 (0)	0 (0)	0 (0)	
Intracerebral hemorrhage/Contusion				**0.01**				**0.02**
No	63 (54.8)	62 (58.5)	1 (11.1)		63 (55.8)	53 (61.6)	10 (37.0)	
Yes	52 (45.2)	44 (41.5)	8 (88.9)		50 (44.2)	33 (38.4)	17 (63.0)	
Unknown	0 (0)	0 (0)	0 (0)		0 (0)	0 (0)	0 (0)	
Subarachnoid hemorrhage				0.21				0.42
No	88 (76.5)	83 (78.3)	5 (55.6)		86 (76.1)	67 (77.9)	19 (70.4)	
Yes	27 (23.5)	23 (21.7)	4 (44.4)		27 (23.9)	19 (22.1)	8 (29.6)	
Obliteration of the third ventricle or basal cistern				**0.003**				**0.04**
No	81 (70.4)	79 (74.5)	2 (22.2)		79 (69.9)	65 (75.6)	14 (51.9)	
Yes	33 (28.7)	26 (24.5)	7 (77.8)		33 (29.2)	20 (23.3)	13 (48.2)	
Unknown	1 (0.9)	1 (0.9)	0 (0.0)		1 (0.9)	1 (1.2)	0 (0.0)	
Nonevacuated mass lesion				**0.02**				**0.01**
No	106 (92.2)	99 (93.4)	7 (77.8)		104 (92.0)	82 (95.4)	22 (81.5)	
Yes	5 (4.4)	3 (2.8)	2 (22.2)		5 (4.4)	1 (1.2)	4 (14.8)	
Unknown	4 (3.5)	4 (3.8)	0 (0.0)		4 (3.5)	3 (3.5)	1 (3.7)	
Abstracted Marshall Class				**0.001**				**0.01**
1	4 (3.5)	4 (3.8)	0 (0.0)		4 (3.5)	4 (4.7)	0 (0.0)	
2	59 (51.3)	57 (53.8)	2 (22.2)		57 (50.4)	47 (54.7)	10 (37.0)	
3	18 (15.7)	13 (12.3)	5 (55.6)		18 (15.9)	10 (11.6)	8 (29.6)	
4	0 (0.0)	0 (0)	0 (0)		0 (0)	0 (0)	0 (0)	
5	25 (21.7)	25 (23.6)	0 (0.0)		25 (22.1)	21 (24.4)	4 (14.8)	
6	5 (4.4)	3 (2.8)	2 (22.2)		5 (4.4)	1 (1.2)	4 (14.8)	
Unknown	4 (3.5)	4 (3.8)	0 (0.0)		4 (3.5)	3 (3.5)	1 (3.7)	

Bold indicates *p* < 0.05.

AIS, Abbreviated Injury Scale; CT, computed tomography; GCS, Glasgow Coma Scale; GOS-E Peds, Glasgow Outcome Scale-Extended for Pediatrics; ICU, intensive care unit; SD, standard deviation.

Approximately half (49.6%) had hypotension and 28.7% had hypoxia either on admission or during the first ICU day. All patients had CT scan reports at either ICU admission or during ICU day 1. Marshall classifications were estimated to be 3.5% I, 51.3% II, 15.7% III, 21.7% V, and 4.4% VI (Table 1). Nine (7.8%) of 115 patients were deceased by day 14, attributed to neurological (*n* = 7), systemic (*n* = 1), and disability-related (*n* = 1) factors. Eighty-six (74.1%) patients had a favorable outcome (GOS-E Peds 5–8), and 27 (23.3%) had an unfavorable outcome (GOS-E Peds 1–4) at 3 months. Two patients had unknown 3-month GOS-E Peds, so were included in the 14-day mortality analyses only.

### Univariate associations with outcomes using PEGASUS data

Significant univariate predictors for 14-day mortality were lower admission GCS motor (*p* = 0.001), fewer reactive pupils (*p* = 0.002), compressed or absent basal cisterns (*p* = 0.002), presence of ICH/contusions (*p* = 0.01), obliteration of 3^rd^ ventricle or basal cistern (*p* = 0.003), nonevacuated mass lesion (*p* = 0.02), and abstracted Marshall classification (*p* = 0.001, Table 1).

Significant univariate predictors for unfavorable 3-month GOS-E Peds were lower admission GCS motor (*p* = 0.01), hypotension (*p* = 0.005), compressed or absent basal cisterns (*p* = 0.01), lack of EDH (*p* = 0.01), presence of SDH (*p* = 0.005), presence of ICH/contusion (*p* = 0.02), obliteration of the third ventricle or basal cistern (*p* = 0.04), nonevacuated mass lesion (*p* = 0.01), and abstracted Marshall classification (*p* = 0.01, Table 1).

### Core and full PEGASUS models for 14-day mortality

Table 2 shows that in the core model, admission GCS motor 2–3, hypotension, and abnormal pupil response were associated with higher odds of 14-day mortality. In the full model, admission GCS motor 2–3, hypotension, and basal cistern compression on CT were associated with higher odds of 14-day mortality.

**Table 2.** Univariate Clinical and CT Factors Associated with 14-Day Mortality and Unfavorable 3-Month GOS-E Peds^a^ After Pediatric Severe TBI Using PEGASUS Model

	Odds ratio (95% confidence interval)			
	14-day mortality		Unfavorable 3-month GOS-E Peds	
Clinical variables	Core model	Full model	Core model	Full model
Age	1.10 (0.99–1.22)	1.15 (0.93–1.42)	1.05 (0.98–1.12)	1.05 (0.98–1.12)
Admission or Day 1 ICU hypotension	**50.55 (2.24–1141.66)**	**947.59 (5.13–175182.50)**	**6.47 (1.23–34.13)**	**18.31 (3.18–105.39)**
Admission GCS motor				
1	2.50 (0.50–12.54)	7.13 (0.19–270.31)	**3.14 (1.04–9.44)**	3.50 (0.81–15.16)
2–3	**24.49 (3.35–178.88)**	**227.77 (4.08–12718.22)**	**11.07 (2.14–57.34)**	**117.18 (5.53–2482.80)**
4–6	Ref	Ref	Ref	Ref
Abnormal pupil response	17.53 (2.22–138.45)	10.32 (0.34–310.59)	2.63 (0.58–11.86)	1.19 (0.12–12.18)
CT variables				
Nonevacuated mass lesion		6.48 (0.99–42.31)		NS
Compressed basal cistern		**36.67 (2.41–558.60)**		NS
Subdural hematoma		NS		**5.80 (2.78–12.12)**
Epidural hematoma		NS		**0.09 (0.01–0.66)**
Midline shift >5 mm		NS		**0.08 (0.01–0.88)**
Abstracted Marshall Class				
1–2		NS		Ref
3		NS		**7.08 (1.27–39.64)**
5–6		NS		**38.62 (3.28–454.20)**

Bold indicates *p* < 0.05.

^a^ GOS-${\left(1+x\right)}^{n}=1+\frac{nx}{1!}+\frac{n\left(n-1\right)x^{2}}{2!}+\ldots$ Peds: Glasgow Outcome Scale-Extended for Pediatrics.

CT, computed tomography; GCS, Glasgow Coma Scale; ICU, intensive care unit; NS, not significant and not in final model; PEGASUS, Pediatric Guideline Adherence and Outcomes, Pediatric Guideline Adherence and Outcomes; TBI, traumatic brain injury.

Table 3 shows excellent model performance for both core (AUROC: 0.92; CI: 0.85–0.99) and full (AUROC: 0.96; CI: 0.89–1.00) PEGASUS models for 14-day mortality.

**Table 3.** Model Fit and AUROC for 14-Day Mortality and 3-Month GOS-E Peds Prognostic Models

	Core		Full	
Model	H-L fit, *p*	AUROC	H-L fit, *p*	AUROC
14-day mortality				
PEGASUS	0.99	0.92 (0.85–0.99)	0.35	0.96 (0.89–1.00)
Petroni	<0.0001	0.88 (0.78–0.98)	0.002	0.93 (0.85–1.00)
IMPACT	0.03	0.86 (0.75–0.97)	0.17	0.92 (0.84–1.00)
CRASH	<0.0001	0.85 (0.68–1.00)	NA	NA
3-month GOS-E Peds				
PEGASUS	0.86	0.82 (0.73–0.91)	0.26	0.91 (0.85–0.97)
Petroni	<0.0001	0.77 (0.65–0.89)	<0.0001	0.83 (0.73–0.93)
IMPACT	0.06	0.72 (0.61–0.84)	0.47	0.82 (0.72–0.92)
CRASH	<0.0001	0.68 (0.55–0.81)	NA	NA

AUROC, area under the receiver operating characteristic curve; Core, clinical variables only; CRASH, Corticosteroid Randomization after Significant Head Injury; Full, clinical and computed tomography variables; GOS-E Peds, Glasgow Outcome Scale-Extended for Pediatrics; IMPACT, International Mission on Prognosis and Analysis of Randomized Controlled Trials in TBI; NA, not available due to lack of data; PEGASUS, Pediatric Guideline Adherence and Outcomes.

### Core and full PEGASUS models for 3-month GOS-E Peds

Table 2 shows that in the core model, admission GCS motor of 1 or 2–3, compared with 4–6, and hypotension were associated with higher odds of unfavorable outcome. In the full model, admission GCS motor 2–3, hypotension, SDH, and abstracted Marshall classification of 3 or 5–6 were associated with higher odds of an unfavorable outcome. The presence of EDH and midline shift >5 mm was associated with lower odds of unfavorable outcomes.

Table 3 shows excellent model performance for both core (AUROC: 0.86; CI: 0.73–0.91) and full (AUROC: 0.91; CI: 0.85–0.97) PEGASUS models for 3-month GOS-E Peds.

### Differences across other prognostic models

We compared AUROC across four core (IMPACT, CRASH, Petroni, and PEGASUS), and across three full (IMPACT, Petroni, and PEGASUS) prognostic models for each outcome. There was no difference for either 14-day mortality or 3-month GOS-E Peds.

### Differences between other respective core and full prognostic models

We compared AUROC between core and full models for each of these three models: IMPACT, Petroni, and PEGASUS for each outcome. There was no difference in AUROC for 14-day mortality. However, full IMPACT and PEGASUS models demonstrated better model performance than core models for 3-month prognostication, whereas Petroni model performance achieved borderline significance (Table 4).

**Table 4.** Core Versus Full Model Prediction Comparison for 3-Month GOS-E Peds

Core Versus full 3-month GOS-E Peds model comparison		
Model	Chi-square	*p* value
PEGASUS	5.91	0.015*
Petroni	3.73	0.0535
IMPACT	6.42	0.011*
CRASH	NA	NA

CRASH, Corticosteroid Randomization after Significant Head Injury; GOS-E Peds, Glasgow Outcome Scale-Extended for Pediatrics; IMPACT, International Mission on Prognosis and Analysis of Randomized Controlled Trials in TBI; NA, not available due to lack of data; PEGASUS, Pediatric Guideline Adherence and Outcomes.

### Model fit

Table 3 shows that PEGASUS achieved good model fit for core and full modes and both outcomes. IMPACT achieved good model fit for the full model only for both 14-day mortality and 3-month GOS-E Peds.

## Discussion

We evaluated if a locally derived South American pediatric-specific early PEGASUS prognostication model with clinical variables alone (core model) outperforms the PEGASUS model with both clinical and CT variables (full model) for 14-day mortality and 3-month outcomes. We also validated select established models (IMPACT, CRASH, Petroni) with PEGASUS data. Main findings are that: (1) the PEGASUS model shows good model fit and excellent prediction of 14-day and 3-month outcomes postpediatric sTBI, (2) there was no difference in core or full model performance for 14-day mortality across models, and (3) full IMPACT and PEGASUS models performed significantly better than respective core models for 3-month GOS-E Peds, whereas the Petroni model achieved borderline significance. Data suggest that the PEGASUS core model can be used for early prognostication of 14-day mortality and 3-month functional outcomes in pediatric sTBI in South America if CT variables are not available at ICU admission. While we validated some models with PEGASUS data and demonstrated good model performance for IMPACT, CRASH, and Petroni, external validation of PEGASUS model with larger pediatric sTBI datasets from regions most affected by sTBI is warranted.

Prior TBI prognosis algorithms are often based primarily on CT information,^13–15^ which may not always be available, particularly in LMICs.^7^ Other TBI prognosis models based on adult clinical data^4,6^ may not predict pediatric sTBI outcomes. Similar to prior studies and aside from EDH, our study found hypotension, GCS motor of 2–3, and intracranial hemorrhage predict unfavorable outcomes. In adjusted analyses, the full PEGASUS model found lower odds of unfavorable 3-month outcomes with CT midline shift >5 mm, potentially due to worse outcomes for patients with a similar initial GCS and pupillary response from diffuse parenchymal injuries that do not cause mass effect or from other confounding factors. Overall, data suggest that ICU clinical variables alone (core model) can be used to predict both 14-day mortality and 3-month GOS-E Peds in South America if CT data are not available in pediatric sTBI.

Differences in social and economic contexts, mechanism of injury, prehospital care, and withdrawal of life-sustaining therapies in LMICs may impact TBI prognosis.^1,7^ The CRASH dataset shows lower GCS to be a stronger predictor of poor outcome in LMICs than in HICs.^6^ The IMPACT dataset was mostly from patients in HICs, and when externally validated with CRASH trial patients, the IMPACT model performed better in predicting outcomes for patients from HICs.^4^ Although based on adults, we thought the Petroni model^7^ may more closely reflect mortality outcomes for pediatric patients than the IMPACT or CRASH models because the Petroni model is based on a population in which withdrawal of life-sustaining therapies is rare, making it more closely resemble care provided to pediatric sTBI patients; however, we did not find this to be true. While there was no difference found between the four core and three full models examined in this study, PEGASUS model development allows for external validation with other pediatric-only sTBI data from South America and other geographical contexts to understand the role country or region play in pediatric sTBI prognostication model performance. Whether or not all LMICs or HICs need local models should also be studied.

The IMPACT and CRASH prognostic models were derived from large and complex TBI datasets and include mostly older children and adults,^4–6,10^ but pediatric-specific TBI models may be needed for prognostication. Studies examining the utility of adult TBI prognostic models in pediatric patients are important because the use of adult models could either over- or underestimate risk of mortality or unfavorable outcome in children, and the accuracy of adult models may be improved by reweighting or adding variables.^16–20^ Liesemer et al. reported that higher Rotterdam scores underestimated and lower Rotterdam scores overestimated mortality in pediatric patients compared with adults; model refinement that included GCS, Rotterdam score, mechanism of injury, and ISS improved discrimination, calibration, and fit.^20^ Although there was no statistical difference in model performance between tested models and PEGASUS, external validation of the PEGASUS model with larger numbers of young children is warranted before concluding that pediatric-specific prognostic models in sTBI are not needed.

Limitations include potential residual confounders, inability to review original CT scans to determine Marshall classification, and lack of outcomes beyond 3 months. The use of an adapted classification system assumed that if a patient had certain intracranial lesions (SDH, EDH, or ICH) and did not have surgery, they could be classified into the nonevacuated mass lesion classification (VI) group which in the original system required that the lesion be >25 mL. No patients were classification IV (midline shift without a mass lesion), as anyone who had EDH, SDH, or ICH would have been classified as either evacuated or nonevacuated mass lesion regardless of size. Because most PEGASUS patients were from Argentina, the study generalizability to other LMICs or HICs is not certain. The low mortality rate limited the value of the prognosis model for 14-day mortality; prehospital care and transfer outcomes may have resulted in survival bias. Although there was no difference between core and full models for 14-day mortality, there were few deaths. The number of outcome events is small, and there is a risk of overfitting given the number of variables tested and used in the model. We did not externally validate our model on external data, nor did we use ICU data beyond day 1. We did not test the IMPACT Lab model because PEGASUS did not collect laboratory data. Strengths of this study include a robust dataset of risk factors and outcomes in pediatric sTBI patients from South America leveraging locally collected data with no interventions, inclusion of young children, age-specific hypotension definition of hypotension, and derivation of a pediatric-specific sTBI model that can be externally validated and guide future attempts at sTBI prognostication across socioeconomic contexts.

## Conclusions

We developed the first pediatric sTBI prognostication model from South American data. Despite the small sample size, the locally derived PEGASUS early prediction model shows good model fit, and excellent prediction of 14-day and 3-month outcomes. Similar to other tested models, the addition of first head CT scan variables to clinical variables from ICU day 1 improved model performance for the PEGASUS models for prediction of early outcomes postpediatric sTBI. Additional study of the PEGASUS model in LMICs for early pediatric sTBI prognostication is warranted.

## Transparency, Rigor, and Reproducibility

The primary study was preregistered at clinicaltrials.gov (**NCT03896789)**. This secondary data analysis was not formally preregistered. The sample size of 115 subjects included in this analysis was based on the eligible admitted to the study sites during the preimplementation baseline stage (September 1, 2019–July 13, 2020, prior to site randomization to primary intervention) patients who consented to data collection of usual care indicators. Data were extracted from medical records; 127 potential participants were screened and 116 were consented to data collection (five patients rejected consent, six died between screening and completion of consent process, and one patient was missing both outcome variables). This retrospective data analysis used deidentified data and selected indicators relevant to build our own prediction model for outcomes after sTBI and to validate the CRASH, IMPACT, and Petroni et al. prognosis models. Investigators did not have access to participant codes linking to identifying information. No individual patient analyses were done and neither participants nor hospital site staff were given a patient prognosis based on model fits. STATA Statistical Software Release 16 (StataCorp LLC, College Station, TX, USA) was used for all analyses. The Strengthening the Reporting of Observational studies in Epidemiology (STROBE) checklist for this manuscript is provided in Table 5. The key inclusion criteria and outcome evaluations are established standards. The indicators, deidentified data, and analytic code used in this analysis may be requested from the authors. The full dataset for the research study will be made available in FITBIR (https://fitbir.nih.gov) after the associated primary outcome article is published.

**Table 5.** STROBE Statement: Checklist of Items That Should be Included in Reports of Observational Studies

	Item no.	Recommendation	Page no.
Title and abstract	1	(*a*) Indicate the study’s design with a commonly used term in the title or the abstract	2
		(*b*) Provide in the abstract an informative and balanced summary of what was done and what was found	2
Introduction			
Background/rationale	2	Explain the scientific background and rationale for the investigation being reported	3
Objectives	3	State-specific objectives, including any prespecified hypotheses	4
Methods			
Study design	4	Present key elements of study design early in the paper	5
Setting	5	Describe the setting, locations, and relevant dates, including periods of recruitment, exposure, follow-up, and data collection	5
Participants	6	(*a*) *Cohort study*—Give the eligibility criteria, and the sources and methods of selection of participants. Describe methods of follow-up *Case*–*control study*—Give the eligibility criteria, and the sources and methods of case ascertainment and control selection. Give the rationale for the choice of cases and controls *Cross-sectional study*—Give the eligibility criteria, and the sources and methods of selection of participants	5
		(*b*) *Cohort study*—For matched studies, give matching criteria and number of exposed and unexposed *Case*–*control study*—For matched studies, give matching criteria and the number of controls per case	n/a
Variables	7	Clearly define all outcomes, exposures, predictors, potential confounders, and effect modifiers. Give diagnostic criteria, if applicable	5–6
Data sources/ measurement	8^a^	For each variable of interest, give sources of data and details of methods of assessment (measurement). Describe comparability of assessment methods if there is more than one group	5–6
Bias	9	Describe any efforts to address potential sources of bias	n/a
Study size	10	Explain how the study size was arrived at	5
Quantitative variables	11	Explain how quantitative variables were handled in the analyses. If applicable, describe which groupings were chosen and why	7–8
Statistical methods	12	(*a*) Describe all statistical methods, including those used to control for confounding	7–8
		(*b*) Describe any methods used to examine subgroups and interactions	n/a
		(*c*) Explain how missing data were addressed	8
		(*d*) *Cohort study*—If applicable, explain how loss to follow-up was addressed *Case*–*control study*—If applicable, explain how matching of cases and controls was addressed *Cross-sectional study*—If applicable, describe analytical methods taking account of sampling strategy	n/a
		(*e*) Describe any sensitivity analyses	n/a
Results			
Participants	13^a^	(a) Report numbers of individuals at each stage of study—e.g., numbers potentially eligible, examined for eligibility, confirmed eligible, included in the study, completing follow-up, and analyzed	8, 27
		(b) Give reasons for nonparticipation at each stage	27
		(c) Consider use of a flow diagram	27
Descriptive data	14^a^	(a) Give characteristics of study participants (e.g., demographic, clinical, social) and information on exposures and potential confounders	8–9, 20–21
		(b) Indicate number of participants with missing data for each variable of interest	8–9, 20–21
		(c) *Cohort study*—Summarize follow-up time (eg, average and total amount)	n/a
Outcome data	15^a^	*Cohort study*—Report numbers of outcome events or summary measures over time	n/a
		*Case*–*control study—*Report numbers in each exposure category, or summary measures of exposure	n/a
		*Cross-sectional study—*Report numbers of outcome events or summary measures	9, 20, 22
Main results	16	(*a*) Give unadjusted estimates and, if applicable, confounder-adjusted estimates and their precision (eg, 95% confidence interval). Make clear which confounders were adjusted for and why they were included	10–11, 22
		(*b*) Report category boundaries when continuous variables were categorized	n/a
		(*c*) If relevant, consider translating estimates of relative risk into absolute risk for a meaningful time period	n/a
Other analyses	17	Report other analyses done—e.g., analyses of subgroups and interactions, and sensitivity analyses	11, 23
Discussion			
Key results	18		Summarize key results with reference to study objectives	12
Limitations	19		Discuss limitations of the study, taking into account sources of potential bias or imprecision. Discuss both direction and magnitude of any potential bias	14–15
Interpretation	20		Give a cautious overall interpretation of results considering objectives, limitations, multiplicity of analyses, results from similar studies, and other relevant evidence	12, 15
Generalizability	21		Discuss the generalizability (external validity) of the study results	14–15
Other information				
Funding	22		Give the source of funding and the role of the funders for the present study and, if applicable, for the original study on which the present article is based	17

An Explanation and Elaboration article discusses each checklist item and gives methodological background and published examples of transparent reporting. The STROBE checklist is best used in conjunction with this article (freely available on the Web sites of PLoS Medicine at http://www.plosmedicine.org/, Annals of Internal Medicine at http://www.annals.org/, and Epidemiology at http://www.epidem.com/). Information on the STROBE Initiative is available at www.strobe-statement.org.

^a^ Give information separately for cases and controls in case–control studies and, if applicable, for exposed and unexposed groups in cohort and cross-sectional studies.

## Abbreviations Used

AIS: Abbreviated Injury Scale
AUROC: area under the receiver operating characteristic curve
CI: confidence interval
CRASH: Corticosteroid Randomization after Significant Head Injury
CT: computed tomography
EDH: epidural hematoma
GCS: Glasgow Coma Scale
GOS-E Peds: Glasgow Outcome Scale-Extended for Pediatrics
HIC: high-income country
ICH: intracerebral hemorrhage
ICU: intensive care unit
IMPACT: International Mission on Prognosis and Analysis of Randomized Controlled Trials in TBI
LMIC: low- and middle-income country
PEGASUS: Pediatric Guideline Adherence and Outcomes
SBP: systolic blood pressure
SD: standard deviation
SDH: subdural hematoma
sTBI: severe traumatic brain injury
STROBE: Strengthening the Reporting of Observational studies in Epidemiology
TBI: traumatic brain injury
